# Assessing health workers’ revenues and coping strategies in Nigeria — a mixed-methods study

**DOI:** 10.1186/1472-6963-13-387

**Published:** 2013-10-04

**Authors:** Ngozi Akwataghibe, Dulani Samaranayake, Christophe Lemiere, Marjolein Dieleman

**Affiliations:** 1 ENAULD Health Research and Services, The Hague, Netherlands; 2 Department of Community Medicine, Faculty of Medicine, University of Colombo, Colombo, Sri Lanka; 3 The World Bank, Africa Region, Africa; 4 Royal Tropical Institute, Amsterdam, Netherlands; 5 Koningin Julianaweg 101, 2264, BC Leidschendam, The Netherlands

**Keywords:** Salaries, Coping mechanisms, Health workers, Mixed methods, Randomized response technique

## Abstract

**Background:**

The setting of realistic performance-based financing rewards necessitates not just knowledge of health workers’ salaries, but of the revenue that accrues from their additional income-generating activities. This study examined the coping mechanisms of health workers in the public health sector of Nasarawa and Ondo states in Nigeria to supplement their salaries and benefits; it also estimated the proportionate value of the revenues from those coping mechanisms in relation to the health workers’ official incomes.

**Methods:**

This study adopted a mixed-methods approach, consisting of semi-structured interviews, a review of policy documents, a survey using self-administered questionnaires, and the randomized response technique (RRT). In all, 170 health workers (86 in Ondo, 84 in Nasarawa) participated in the survey. In-depth interviews were conducted with 24 health workers (12 per state) and nine policy makers from both states.

**Results:**

The health workers perceived their salaries as inadequate, though most policy makers differed in this assessment. There appeared to be a considerable expenditure–income disparity among the respondents. Approximately 56% (n = 93) of the study population reported having additional earning arrangements: most reported non-medical activities such as farming and trading, but private practice was also frequently reported.

Half of the respondents with additional earning arrangements stated that their income from those activities was the equivalent of half or more of their monthly salaries. Specifically, 35% (n = 32) said that they earned about half of their official monthly salaries and 15% (n = 14) reported earning the same or more than their monthly salaries from these activities. Other coping mechanisms used by the health workers included prioritizing activities that enabled the earning of per diems, collecting informal payments and gifts from patients, and pilfering drugs from facilities.

**Conclusions:**

Predatory and non-predatory mechanisms accounted for the health workers’ additional income. It may be difficult for the health workers to meet their expenses with their salaries and financial incentives; this highlights the need for the regulation of additional earnings and to implement targeted accountability mechanisms. This study indicates the value of using mixed methods when investigating sensitive issues. Future studies of this type should employ mixed methods for triangulation purposes to provide better insight into health workers’ responses.

## Background

Significant progress has been made in the Nigerian government’s commitment to the health sector and to policy development since the reinstitution of democracy in 1999 [1]. However, at approximately 4% of total expenditure, the government expenditure on health [2] still lags behind the 15% recommended by African leaders at the Abuja Summit in 2000 [3]. Health-care delivery continues to be a challenge in Nigeria, and the country is still not on track to achieve many of the health-related Millennium Development Goals. Therefore, there is a need to promote greater progress toward attaining global targets.

In Nigeria, as in many other low- and middle-income countries (LMICs), the poor performance of public health systems can be attributed to a number of factors. These range from poor or inadequate funding of the health sector, shortages in the health workforce, and a limited capacity in health management within the country to problems that extend beyond the health sector [4,5], such as weak governance and poverty among certain populations.

The Nigeria State Health Investment Project intends to introduce resource allocation based on results and to pilot a performance-based financing (PBF) mechanism in three Nigerian states. Output-based payments, which are a main strategy in performance-based approaches, have been employed in several low-income countries, Rwanda and Burundi being typical examples [6].

A key issue is that the setting of realistic rewards for health workers in the context of PBF demands knowledge about the real income of health workers. This necessitates knowledge not just of salaries, but also of the revenue that accrues from additional income-generating activities. Public salaries in many countries are low, and though the financial aspect is only one of many that motivate workers, it is a crucial one [7]. It is known that health workers’ responses to unfavorable working conditions and inadequate salaries are manifested in the form of various coping mechanisms to supplement salaries. Income supplementation by health workers allows them to live closer to their desired standard of comfort, and it may have the arguable advantage of keeping qualified personnel in the public service. These mechanisms may include the following: moonlighting during work hours, either through medical work (i.e., private practice) or non-medical work [7-9]; concentrating on donor-funded activities that give access to per diems or allowances [7,8,10]; or the misuse of their positions for misappropriation [7], such as pilfering drugs, which can provide a substantial means of revenue for health workers when sold to patients or on the parallel drug market [9,11]. Not all these practices present a conflict of interest or are illegal, but they do affect the functioning of health services. Some predatory mechanisms (such as drug pilfering, collecting illegal payments from patients, and moonlighting activities that divert patients to private practice) constitute an outright financial barrier to access to health-care and reduce public trust; other non-predatory mechanisms, such as giving priority to per diem activities, constitute more of a competition for time [7] and indirectly have a negative impact on access to care.

Few studies have assessed the value of revenues gained from such coping strategies. Questions about the coping strategies of health workers to supplement their income are sensitive, and it is difficult to obtain truthful answers. For instance, the misuse of health workers’ access to drugs though rarely openly acknowledged, is considered a widespread practice [7]. In a study conducted in Uganda, the average Ugandan health facility drug leakage was found to be as high as 78%, and the resale of drugs was identified as the single highest income earner for most units. Both health workers and management teams were involved in this activity [11]. A study of a sample of health workers in Mozambique and Cape Verde confirmed the misuse of access to drugs by health workers as a major coping strategy [9]. That study found that different categories of health workers misappropriated drugs in different ways, but that doctors used the most diverse methods. The study noted a conflict in health workers’ self-image that arose in terms of their perception of what an honest public servant should be and the difficult living situations they faced, which made them betray that image. The results also showed that despite the difficulties health workers faced in many countries, these illegal acts had not been internalized as a norm, and that policies resulting in an improvement in the state of affairs would be welcomed by health personnel.

To improve the existing situation, alternative strategies to conventional input-based methods may have to be implemented to improve the performance of the health sector and to help it achieve its goals. There is a need for approaches that maximize the use of available resources to achieve increased productivity and quality performance from the available workforce [6]. One of the major challenges is the fact that salaries are fixed, and are not linked to the provision of services; there are few incentives to motivate health workers to be responsive to the communities for which they provide services [8]. Indeed, the question of how to structure payments in the sector to encourage better performance is one that is currently generating considerable interest [12,13]. PBF schemes have received great attention as an innovative approach to improve the use and quality of health-care services as well as the efficiency of the health system in LMICs.

Two states in Nigeria were chosen for this study—Nasarawa and Ondo. These states, which were used as pilots for the institution of PBF, have diverse characteristics in terms of population, number of health workers and available health worker types.

### Nasarawa state

Nasarawa State is in the north central part of Nigeria. It has a population of approximately 1.9 million and comprises 13 local government areas (LGAs). The state has 880 primary health facilities, 19 secondary public health facilities and two tertiary health facilities [14]. Human health resources in the public sector consist of 215 doctors, 1021 nurses and midwives, 55 community health officers (CHOs), and 3365 community health extension workers (CHEWs) [14].

### Ondo state

Ondo State is in the southwestern part of the country. It has a population of around 3.9 million and is made up of 18 LGAs [15]. The state has about 800 primary health facilities, 16 secondary public health facilities, and six tertiary ^a^ health facilities [16]. Human health resources in the public sector include approximately 60 senior doctors (consultants), 190 medical officers, 1475 nurses/midwives, 185 CHOs, and 1152 CHEWs [17]. The provision of health services in the state is free for pregnant women and for children aged up to 5 years. The state implements a national health insurance scheme in 12 LGAs.

This paper presents the results of a baseline study on income and coping mechanisms of health workers to supplement their public sector salaries in these two states, to identify opportunities for rewarding health workers in the context of PBF. The present study is part of a wider investigation involving motivation and performance management issues. The focus of the study presented here was directed at answering the following specific study questions. What are the opinions of the health workers regarding their salaries and benefits? What coping mechanisms are adopted by different types of health workers—doctors, nurses, CHOs, and CHEWs—in dealing with income-related issues? What is the proportionate value of the revenues from those coping mechanisms in relation to the health workers’ official income? What are the opinions of key experts in the ministries of health and local governments regarding health workers, and which policies influence their views on salaries and benefits?

## Methods

This was a mixed-method study, which focused on four critical types of workers in primary and secondary health-care (nurses/midwives, CHOs, and CHEWs) and secondary and tertiary care (doctors).

### Data collection methods

The quantitative aspect was addressed by the use of a questionnaire made up of the following two components.

Self-administered section on health workers’ compensation - This part of the questionnaire mainly addressed the health workers’ salaries and their additional sources of revenue, including the amount generated, their household expenditure, and satisfaction with their salaries and living conditions.

The randomized response technique (RRT)^b^ section- The RRT is a survey methodology that allows respondents to answer sensitive questions truthfully without invading their privacy [18]. The RRT explored the different coping mechanisms of health workers, including the prioritization of activities that enabled the earning of per diems, selling drugs to patients, diverting patients to private practice, and pilfering drugs or supplies from health facilities. This part of the study included a series of 10 randomized questions: seven sensitive questions interspersed with three less sensitive questions.

The qualitative aspect was addressed by means of secondary data and semi-structured interviews with health workers and key informants in the health ministries and local governments. The semi-structured interviews were carried out in each state with three individuals from each health worker type. The interviews explored their perceptions of the adequacy of their salaries and the need to supplement their income, including their coping strategies. Semi-structured interviews with key informants from health ministries and local government examined the setting of health workers’ salaries, their satisfaction with their salaries, the need for health workers to supplement their income, and the various options open to them in this regard. Secondary data were obtained from policy documents on salary scales and setting salaries.

### Study population and sampling

LGAs from the two states were purposively selected using maximum variation sampling based on criteria of geographic features: LGAs in urban, semi-urban, and rural areas; and the availability of a mixture of health facilities with different types of health workers. Two LGAs were chosen from Ondo State—Akure South and Ifedore; three were chosen from Nasarawa State—Nasarawa Eggon, Doma, and Lafia.

The facilities were purposively selected based on the availability of the required health worker types. Priority was given to primary health facilities in the selection of nurses/midwives, CHOs, and CHEWs. Secondary health facilities were chosen based on the availability of doctors and nurses. In Nasarawa, a tertiary health facility was also selected to recruit the doctor population because of the lack of doctors in the secondary facilities.

In all, 170 health workers (86 and 84 health workers from Ondo and Nasarawa, respectively) were recruited for the study; however, the data for only 165 were analyzed. Five incomplete questionnaires were excluded. The mixture of health worker types varied slightly between the two states: there were 21 doctors, 22 nurses/midwives, 22 CHOs, and 21 CHEWS from Ondo compared with 20 doctors, 22 nurses/midwives, 20 CHOs, and 22 CHEWs in Nasarawa. Random systematic sampling was carried out to recruit all health worker types with the exception of CHOs in Ondo and doctors in Nasarawa. Convenience sampling was conducted for the latter two health worker types owing to their limited numbers. The study recruited all health workers who were available at the facilities, whereas those away on study leave or who were absent through illness were excluded. The response characteristics of the study participants are detailed in Table 1.

**Table 1:** Response characteristics of study participants (n = 165)

**Response characteristic**	**Nasarawa**		**Ondo**	
	**N**	**%**	**N**	**%**
Total recruited	84	-	86	-
Responded	80	95.2^a^	85	98.8^a^
Non-Reticent by standard criteria*	76	95.0	69	81.2
Non-Reticent by modified criteria**	41	51.3	28	32.9
In-depth interviews	12	-	12	-

* After excluding participants answering ‘No’ to at least all six sensitive questions in the RRQ and ‘Yes’ to one or more of the less sensitive questions (questions 1, 2, and 10) or question no. 3, which was also perceived as being less sensitive.

** After excluding those responding ‘No’ to five or more questions in the RRQ.

^a^ Out of total recruited.

For the qualitative component, 24 health workers (12 in each state) were selected from the respondents using maximum variation sampling, based on the type of health worker (three individuals per type) and years of work experience (less than 5 years and 5 years or more). The health workers were based at six health facilities in Ondo and nine facilities in Nasarawa.

Nine policy makers from ministries of health and local government were also selected for interviews. They included the following: a health commissioner, four directors of primary health-care of the LGAs selected for the study, one permanent secretary of health, a chief medical director of a specialist hospital, and a director and deputy director at the ministries of health.

### Data analysis

#### Quantitative analysis

Quantitative data were entered using SPSS version 19 software and checked for consistency using frequency distribution and manual checking. Data were then cleaned by referring back to the original questionnaires. Standard descriptive statistical methodologies were used to capture the socio-demographic and household characteristics, salary and other incentives, and additional earning arrangements. Comparisons among states and health worker categories were made using chi-square tests and non-parametric tests as appropriate.

#### Process and analysis of randomized response questionnaire (RRQ)

The RRQ was used to identify the reticent respondents. The process is as follows. The participants are instructed to toss a coin at each question and answer depending on the results of the coin toss. If the participant obtains heads, the question is answered as yes, irrespective of the answer the participant would actually give. If the participant obtains tails, the question is answered honestly. The number of yes answers obtained per question therefore consists of those who obtained heads after tossing the coin and those who answered yes to engaging in sensitive behavior when landing tails in the coin toss. Assuming the proportion of participants obtaining heads at the coin toss to be 0.5, the proportion of participants in the sample actually engaging in sensitive behavior (*P)* is given by the following:

$$P=2P_{Y}–1$$

where *P*_*Y*_ is the proportion of participants answering yes to the particular sensitive question.

#### Identification of reticent respondents in the RRQ

The additional earning strategies assumed to be practiced by health workers include behavior and strategies that are considered unethical or unacceptable by societal standards. Therefore, there was a high possibility that participants would be reluctant to reveal these practices. The RRQ was thus used to address this issue. The RRQ was expected to achieve two objectives:

1. Encourage candor on the part of participants when answering questions regarding sensitive behavior by means of the coin-toss method.

2. Identify possible participant reticence using a combination of sensitive and less sensitive questions.

Questions 1, 2, and 10 were less sensitive questions, and they were included to identify reticence in the sample. It was assumed that reticent participants would answer no to all questions, which is highly unlikely to occur with the coin-toss method. Alternatively, it was supposed that reticent participants would answer yes to only the less sensitive questions (1, 2, and 10) and answer no to all the sensitive questions. Hence, it was assumed that reticent respondents could be identified by examining the pattern in answering the questions. A close examination of the answering pattern showed that question 3 (“I give priority to job activities that will enable me to earn per diems”) was also perceived as relatively less sensitive by the participants. Therefore, in the analysis of the RRQ, “possibly reticent” participants were excluded. The reticent participants were initially defined as follows:

-Participants answering no to all questions (it was noted that this group may either not have understood the process or did not take it seriously; three participants were excluded based on this criterion).

-Participants answering yes to only one question and no to all other questions.

-Participants answering no to all six sensitive questions and yes to one or more of the less sensitive questions (questions 1, 2, and 10) or the relatively less sensitive question 3.

Once reticent subjects were excluded, the sample consisted of 145 subjects. However, analysis of these subjects resulted in many unrealistic percentages, showing that a high degree of reticence remained within the sample. The criteria for reticence were then expanded. For the purposes of this study, reticent participants were defined as those who answered no to five or more questions. Once reticent subjects were excluded, the sample consisted of 69 subjects. These non-reticent subjects were then analyzed.

#### Qualitative analysis

A combined technique of deductive and inductive analysis was carried out. Transcripts were read and coded, and data were manually extracted using a data analysis matrix. This matrix consisted of columns with pre-identified codes according to the research objectives and themes of the interview guide. During the coding of the transcripts and the data extraction, new themes emerged, which were added to the data analysis matrix. Subsequently, answers from the two states and from the different types of health workers were compared and contrasted. This was first done individually and the team then discussed their findings and compared their analysis, reflecting on the findings. The states and health worker types were analyzed together and separately to enable the identification of both similarities and differences in terms of views and experience.

### Ethical considerations and quality assurance

This study was ethically approved by the National Primary Health Care Development Agency, Nigeria. Informed consent was obtained before administering the questionnaires and conducting the interviews. Participation was on a voluntary basis. Only the study teams had access to the collected data, and in the reporting all links to individuals or facilities were removed.

In view of the sensitive nature of the study subject matter, in that it explored some predatory and illegal practices among health workers, a variety of methods and sources were used to allow triangulation of the information: semi-structured interviews, self-administered questionnaires, and a RRT. The RRT specifically aimed to identify reticent respondents. Different types of health workers and different policy makers were interviewed.

## Results

### Socio-demographic characteristics of respondents

Both states had a predominantly experienced workforce, with only about 19% (n = 32) of the respondents having work experience of 5 years or less. Of the study population, 66% (n = 108) were female, and most (83%, n = 137) of the respondents were married. Approximately 46% (n = 75) of the respondents worked in rural areas while 18% (n = 29) worked in semi-urban areas. The majority (69%; n = 114) worked in primary health facilities. Details of the proportion of respondents from the two states and the facilities as well as the basic socio-demographic characteristics of the sample are presented in Table 2.

**Table 2:** Basic socio-demographic and work-related characteristics of the sample according to state

**Characteristic**		**Nasarawa**		**Ondo**		**Total**	
		**n**	**%**	**n**	**%**	**n**	**%**
Setting	Urban	19	23.8	42	49.4	61	37
	Rural	56	70.0	19	22.4	75	45.5
	Semi urban	5	6.3	24	28.2	29	17.6
							
Type of facility	Basic health clinic	53	66.3	30	35.3	83	50.3
	Comprehensive primary health clinic	0	0	25	29.4	25	15.2
	Model primary health clinic	5	6.3	1	1.2	6	3.6
	General / Secondary hospital	3	3.8	28	32.9	31	18.8
	Tertiary hospital	19	23.8	1	1.2	20	12.1
							
Work experience	5 years or less	19	23.8	13	15.3	32	19.4
	6 – 10 years	17	21.3	11	12.9	28	17.0
	11 – 15 years	14	17.5	14	16.5	28	17.0
	16 – 20 years	16	20.0	14	16.5	30	18.2
	21 – 25 years	10	12.5	5	5.9	15	9.1
	>25 years	4	4.9	28	33.0	32	19.4
							
Gender	Female	41	51.3	67	78.8	108	65.5
	Male	39	48.8	18	21.2	57	34.5
							
Marital status	Single	15	18.8	6	7.1	21	12.7
	Married	61	76.3	76	89.4	137	83.0
	Other*	4	5.1	3	3.5	7	4.2
							
**Total**		80	100	85	100	165	100

For the qualitative study, 24 respondents from the four types of health workers were interviewed from both Nasarawa and Ondo. Three health workers from each type were interviewed in each state. Nurses/midwives, CHOs, and CHEWs were predominantly female (n = 17) and the doctors were predominantly male (n = 4). Almost all the health workers were married, and 17 described themselves as being the main income earners in their households. Three health professionals had less than 5 years’ work experience; the remainder had experience ranging from 5 to 32 years.

### Official remuneration of health workers and its adequacy

Salary structures for health workers in Nigeria are known as CONMESS (consolidated medical salary structure) for medical and dental professionals and CONHESS (consolidated health salary structure) for other health professionals, including nurses/midwives, CHOs, and CHEWs.

Adoption of the salary structure varied in the two states: Nasarawa had 100% implementation of CONMESS and CONHESS, whereas in Ondo the take-up rate was approximately 80%. Nigeria has a vastly decentralized government structure, which consists of three tiers—federal, state, and local governments. This decentralization gives the state a great level of autonomy and the ability to decide what percentage of the salary scale (decided at the federal level) they are able to pay.

A comparison of the salary structure in the two states appears in Table 3, which indicates the implemented CONMESS/CONHESS of the health workers at Grade level (GL) 10, step 1, on the scales. The salaries in Table 3 are presented as combined figures that include all benefits and allowances, such as transport, housing, and rural hardship and hazard allowances. The figures also include duty allowances for doctors and shift duty allowances for the other health worker types.

**Table 3:** Gross salaries of the different health worker types in the two states at Grade Level 10, Step 1

**Cadres**	**Nasarawa**		**Ondo**	
	**NGN**	*USD	**NGN**	*USD
Doctors	212722.00	1350.00	176113.00	1116.00
Nurses/midwives	157846.00	1001.00	126641.00	803.00
CHOs	157846.00	1001.00	126641.00	803.00
CHEWs	157846.00	1001.00	126641.00	803.00

NGN: Nigeria Naira.

*USD: US dollars (Current exchange value of 1 USD is approximately 157.57 NGN).

Entry point into public service for Doctors: CONMESS 10; Nurses/Midwives and CHEWs: 06; CHOs: 07.

Deductions vary, hence net pay varies.

Data on net pay, which would have been preferable, could not be obtained as most health workers did not receive pay slips and were not clear about their net pay.

The pay is low compared with that of health professionals in most developed countries; for example, according to the World Health Organization, in terms of purchasing power parity, Nigerian doctors earn about 25% of what they would earn working in Europe, North America, or the Middle East [19]. However, health workers are considered to be much better paid than other public servants in Nigeria. As an example, a medical officer on GL10 step 1 of the CONMESS scale in Ondo has a gross monthly salary of approximately 180,000 naira, whereas a public servant in another sector on GL 10 step 1 of the enhanced public service salary structure earns a gross salary of around 50,000 naira.

The wage compression ratios for health workers in the two states are within standard limits. Compression is said to occur when there is a small differential in salaries between workers regardless of their skills and experience. Wage compression is calculated as the ratio between the highest and lowest income earners. An acceptable compression ratio by international standards ranges from 6:1 to 7:1 [20]. However, the appropriateness of these figures depends largely on what is politically and constitutionally acceptable in a particular country and what is generally considered socially equitable [20]. The wage compression ratios calculated with the implemented CONHESS scale were 6.9:1 and 6.6:1 in Nasarawa and Ondo, respectively.

The majority of the health workers in the survey did not consider their salaries adequate. Of the 165 health workers, only 22 (13%) were satisfied with their salaries and benefits. An analysis by state and health worker type did not reveal any significant difference in satisfaction levels. All interviewed health workers answered that their salaries were important to provide for their (extended) families. However, health workers also stated that their salaries were insufficient and therefore additional sources of income were required. Additional income was also perceived as necessary because of societal pressure: relatives of health workers usually have high expectations of them. Two respondents in Nasarawa stated that they were required to look after relatives because of their status as health workers: “Whether you like it or not, society always expects a great deal from you, and the fact that society now worships wealth means that you are constantly under that pressure. And if you are not getting what you think is a fair deal from your employers, the onus is now on you; otherwise, other people will think badly of you” (doctor, Nasarawa).

However, the policy makers differed in their opinion. Most key informants (five of nine) interviewed believed that the salary was adequate for the health workers. Many stated that increasing salaries would only motivate staff for a short period and that they would never be satisfied: *“*Well, to a large extent human beings are insatiable. There is no amount that you can pay somebody such that they would not want to do something else” (key informant, Ondo).

The household expenditure pattern was assessed in relation to the household size. The number of people living in the households of the participants ranged from 1 to 23 with a median of 6 (IQR 4–8). Six or more people were reported to live in 56.9% (n = 94) of the households. The total monthly expenditure of the households ranged from 21,000 to 4,245,000 naira, ^c^ with a median of 190,000 (interquartile range [IQR] 114,600–346,000) naira. The per capita household expenditure ranged from 3,287 to 707,500 naira. The median per capita household expenditure was 35,000 (IQR 20,607–66,267) naira. Of the health workers, 73 (44%) had spouses with full-time employment.

There was considerable disparity between the health workers’ salaries and their household expenditure. The total household expenditure was remarkably high compared with the salaries of the health workers. For approximately 38% (n = 62) of the respondents it was between 101% and 200% of their salaries, and for 20% (n = 33), it was 200%–500% (Details are in Additional file 1: Table S1). From the pattern of household expenditure, it was evident that this asymmetry between expenditure and income occurred mainly in terms of household feeding, school fees for children, and in the proportion of income spent on dependents (extended family members). It was found that 15% and 21% of the health workers spent 51%–100% of their salaries on food and education, respectively; 3% and 16% spent over 100% on these items. For dependents, 22% of the health workers spent 21%–50% of their salaries. Details of the household expenditure of respondents as a proportion of their salaries are shown in Table 4.

**Table 4:** Total household expenditure and its components expressed as a percentage of the respondent’s salary

**Percentage category**	**Food**		Education^*^		**Dependents**		**Rent**		^**#**^**Utilities**		**Transport**	
	**n**	**%**	**n**	**%**	**n**	**%**	**n**	**%**	**n**	**%**	**n**	**%**
20 or less	59	35.8	61	37.0	117	71.0	151	91.5	130	78.8	142	86.1
21–50	68	41.2	39	23.6	37	22.4	6	3.7	26	15.8	14	8.5
51–100	25	15.2	34	20.6	5	3.0	3	1.8	5	3.0	4	2.4
Above 100	5	3.0	27	16.4	3	1.8	2	1.2	2	1.2	0	0.0
Missing	8	4.8	4	2.4	3	1.8	3	1.8	2	1.2	5	3.0
Total	165	100	165	100	165	100	165	100	165	100	165	100

*Education refers to termly school fees paid in September. Health workers were asked for their previous month’s expenditure (data were collected in October).

^#^Utilities include water, electricity, fuel for generators, recharge cards for cell phones (communication).

### Approaches to supplementing income

This section focuses on the three types of additional earning arrangements explored in the study: medical, non-medical, and selling drugs. The results obtained from the compensation questionnaire used in the survey and the in-depth interviews of both health workers and policy makers are presented first, followed by the findings from the RRT.

### Medical and non-medical additional earning arrangements

Of the 165 respondents in the survey, 93 (56.4%) indicated that they had medical or non-medical additional earning arrangements. The majority (n = 70, 42.4%) reported non-medical activities, such as farming and trading. Table 5 shows further details of the different types of additional earning arrangements practiced by participants. Table 6 presents details regarding the time spent on additional earning arrangements and the income generated. Nearly 45% (n = 41) of the respondents that had medical and non-medical additional earning arrangements stated that they spent between 3 to 5 hours a day on such activities. Approximately 21% (n = 19) reported that they spent 3–4 days a week and an additional 35% (n = 32) reported spending 5 or more days a week on these activities. Thirty-five percent (n = 32) of respondents with additional arrangements said that they earned the equivalent of half of their official monthly salaries and an additional 15% (n = 14) reported earning the same or more than their monthly salaries from these activities. The remainder (41%; n = 38) reported earning less than half their monthly salaries from their additional earning arrangements. However, comparing the reported additional incomes with the amount of time reportedly spent on such activities suggests that the additional income reported may have been underestimated.

**Table 5:** Prevalence of medical and non-medical additional earning arrangements in the sample of health workers (n = 165)

**Types of additional earning**	**No.**	**Prevalence**^**a**^
**arrangements**	**practicing**	
Not practicing any additional earning arrangement	72	43.6
Private practice, after normal working hours	34	20.6
Private practice, during working hours	07	4.2
Private practice, on call service	20	12.1
Private practice, baby delivery at home	13	7.9
Non-medical business (farming and trading)	70	42.4
Other activities*	01	0.6
All with one or more additional earning arrangement(s)	93	56.4

* HIV treatment and prevention project.

^a^Prevalence is expressed as a percentage of the total sample of 165 health workers.

Note: some of the respondents reported more than one of these activities.

**Table 6:** Characteristics of medical and non-medical additional earning arrangements by state

**Characteristics**	**Total**		**Nasarawa**		**Ondo**	
	**n**	**%**	**n**	**%**	**n**	**%**
**No. of hours (per day) spent on additional earning**						
1 – 2 hours	39	42.4	26	44.8	13	38.2
3 – 5 hours	41	44.5	25	43.1	16	47.1
>5 hours	10	10.9	6	10.3	4	11.8
Not stated	02	2.2	1	1.7	1	2.9
**No. of days (per week) spent on additional earning**						
1 – 2 days	40	43.4	28	48.3	12	20.7
3 – 4 days	19	20.7	12	20.7	7	12.1
≥5 days	32	34.8	17	29.3	15	25.9
Not stated	01	1.1	1	1.7	0	0.0
**Amount earned from additional arrangements**						
Less than half the monthly salary	38	41.3	2	36.2	17	50.0
About half the monthly salary	32	34.8	2	36.2	11	32.4
Same as monthly salary	09	9.8	7	12.1	2	5.9
More than the monthly salary	05	5.4	3	5.2	2	5.9
Not specified	08	8.7	6	10.3	2	5.9
Total	92	100.0	58	100	34	100

There was a significant difference (p = 0.000) between the two states in the additional earning arrangements undertaken by the study population. About 74% (n = 59) of respondents from Nasarawa reported medical and non-medical additional earning arrangements compared with 40% (n = 34) from Ondo. The CHOs in Nasarawa reported the highest level of such activities (100%; n = 20), whereas the doctors in Ondo reported the least (29%, n = 6). Details of the number of health workers in the different professional types and their medical and non-medical additional earning arrangements appear in Table 7.

**Table 7:** Comparison of additional earning arrangements according to health worker type between the two states

**Cadre**	**Nasarawa**			**Ondo**			**Significance**^**a**^
	**Total**	**n**	**%**	**Total**	**n**	**%**	
CHEW	22	15	68.2	23	12	52.2	NS^b^
CHO	20	20	100	21	8	38.1	0.001
N/M	21	17	81	20	8	40	0.007
Doctor	17	7	41.2	21	6	28.6	NS
All cadres	80	59	74	85	34	40	0.000

Note: Some of the health workers have more than one coping strategy.

^a^ Chi-square test.

^b^ NS – Not statistically significant at *p* = 0.05.

The findings from the qualitative aspect of the study provide some insight into these results. During the interviews, respondents from Ondo answered that health workers should not engage in outside health-related business, as this could be perceived as stealing. In Nasarawa, respondents regarded health-related private practice as more acceptable. Three individuals mentioned that especially for doctors and midwives (1x), private practice was a lucrative source of additional income, and one person declared that selling drugs was also very profitable. The reason for this difference in perception may be partly explained by the view expressed by one of the key informants from Nasarawa about private practice: “I think it’s a good thing because we have advocated that if you are a midwife and are off-duty, you can assist in other areas—go to private health clinics and assist there because we have manpower problems. Both government and private facilities suffer from this problem. So if such people can use their time more to assist others, it will improve child delivery” (key informant, Nasarawa).

Nasarawa is perceived as suffering a greater shortage of health workers than Ondo, and the impression is that some illegal practices may be overlooked if health workers’ activities help solve the health needs of the populace. However, of all the respondents interviewed, only one from Nasarawa, a CHO, reported having a private practice. Others in the two states answered that they prescribed for and treated people at home; however, they were not always explicit about whether or not they charged for treatment. Some respondents gave the impression that they did not charge but sometimes received gifts from patients: “When I am at home, people come, but I just write prescriptions for small drugs like Panadol, Flagyl, and malaria drugs, and they go and buy them outside. You see, it’s my contribution. In fact, because of this, I get things from people for free because they appreciate what I am doing. For instance, somebody has said she will buy clothes for my baby when it’s born. So that’s what I get—good things from people” (CHO, Nasarawa).

Even when health workers said that they treated patients for free and received only gifts in return, the dynamics of this practice—especially in terms of the level of obligation felt by the patients to give gifts—remain unclear. It is likely that some respondents did not consider treating patients at home as private practice.

In Nasarawa, respondents answered that visiting patients at home or treating them in the health workers’ homes might include assisting in deliveries. In Nasarawa, home deliveries appeared to be generally accepted both by the community and by health workers. Patients sometimes made payments for these deliveries, and they were reportedly cheaper than the costs incurred in health facilities. However, this was not the case in Ondo, as deliveries there are free in hospitals. It even appeared that there were quite strong opinions against assisting in deliveries at home, and that this was only acceptable in the case of real need:

I: “What about home deliveries?”

R: “No. It is risky. They don’t do it. No, no, no—very, very risky. We advise pregnant mothers to come to the health facility; we don’t deliver them at home” (CHO, Ondo).

In Ondo, respondents were more explicit about the fact that private practice for the health workers (except doctors) was often forbidden—in both states, private practice is legal outside official working hours for doctors in public service. However, a key informant in Ondo stated that private practice was not practiced so much in the state because of free health-care at local government and state levels.

### Selling drugs as an additional earning arrangement

Selling drugs to patients as an additional earning arrangement was explored in the survey. Given the sensitive nature of this behavior, the participants were questioned both directly (asking respondents about their activities) and indirectly (asking about the activities of others) with regard to this practice. With indirect questioning, 30% (n = 50) of the respondents stated that other health workers at their workplace sell drugs to patients; the median estimated monthly amount earned was 75,000 (IQR 30,000–100,000) naira. When questioned directly on this practice, only 15% (n = 25) admitted to selling drugs to patients. The reported monthly amount earned from this practice was much lower than the earning estimated by their colleagues, with a median of 9,000 (IQR 3,000–20,000) naira. Thus, it is likely that the reported figures were underestimated. Additional file 1: Table S2 shows the reported amounts earned by the health workers from selling drugs as an additional arrangement.

Respondents mostly—more so in Ondo than in Nasarawa—answered that they knew of colleagues, or had heard of others, who ran private practices and sold drugs, but that they had no further details. Most respondents (seven of 12 in Nasarawa, six of 12 in Ondo, and four of nine key informants) confirmed that illegal practices took place at work. These mainly involved referring patients to their own clinic and asking for additional money from patients or selling drugs. However, often respondents answered the questions in terms of the practices taking place at “other facilities” or “the facility where I used to work,” and they denied that such practices occurred in the facility where they currently worked.

When asked about drug revolving funds (DRFs) in the facilities and how they worked, key informants in Nasarawa stated that DRFs did not exist in state facilities. The implication was that drugs were not always available in the facilities and had to be purchased by the patients from other places, thereby creating opportunities for health workers that sell drugs. However, in Ondo, key informants answered that some facilities had DRFs, especially those that operated under the national health insurance scheme.

When asked about rules and regulations in place to control illegal practices, the key informants agreed that there were punitive measures, such as warnings or dismissals, if the health workers did not comply with the rules and regulations about private practice. However, these measures were not known to all health workers and did not seem to be implemented in the same way by all the actors: *“*If you abandon your duty and go into private (practice) and we find out, we give you three warnings. If you repeat the practice, we terminate your appointment” (key informant, Nasarawa). “There are rules; the staff regulation says that if you steal, you will be dismissed. That is clear” (key informant, Ondo).

### Reticence in declaring coping mechanisms

The RRT results (Table 8) indicate that most respondents were reticent about their coping strategies to supplement their income. Of the 165 respondents, 96 (58%) were identified as reticent and were excluded from the RRT analysis. In all, 69 (42%) health workers in the sample were identified as “non-reticent” or candid about their coping strategies to supplement income. Reticence was observed to a greater degree in the Ondo sample (59.4%, n = 57) than in the Nasarawa sample (40.6%, n = 39).

**Table 8:** Analysis of the RRT after excluding possible reticent participants (n = 69)

Question**	**Ondo (n = 28)**			**Nasarawa (n = 41)**			**Total non-reticent (n = 69)**		
	**% ‘Yes’**	% Practice*	**P value**	**% ‘Yes’**	% Practice*	**P value**	**% ‘Yes’**	% Practice*	**P value**
**RR1: I sometimes give priority to my relatives when I am treating hospital patients.**	58.5	50.0 (31.5–68.5)	0.014	80.5	61.0 (46.1–75.9)	0.000	78.3	56.6 (44.9–68.3)	0.000
**RR2: I have enough drugs and equipment to do my work.**	75.0	50.0 (31.5–68.5)	0.014	73.2	46.4 (31.1–61.7)	0.005	73.9	47.8 (36.0–59.6)	0.000
RR3: I give priority to my job activities that will enable me earn per-diems.	75.0	57.2 (38.9–75.5)	0.005	78.0	56 (40.8–71.2)	0.001	78.3	56.6 (44.9–68.3)	0.000
RR4: I sometimes go to a patient’s home to treat them for a fee.	46.4	7.2 (16.8–2.4)	0.850	58.5	17.0 (5.5–28.5)	0.349	53.6	7.2 (1.1–13.3)	0.630
RR5: I make more money from my supplementary sources than from my salary.	67.9	35.8 (18.0–53.6)	0.089	63.4	26.8 (13.2–40.4)	0.118	65.2	30.4 (19.5–41.3)	0.016
RR6: I sometimes accept money or gifts from patients to give them priority treatment.	57.1	14.2 (1.3–27.1)	0.571	73.2	46.4 (31.1–61.7)	0.005	66.7	33.4 (22.3–44.5)	0.008
RR7: I have referred patients from the public hospital to my private practice.	53.6	7.2 (2.4–16.8)	0.850	61.0	22.0 (9.3–34.7)	0.212	58	16.0 (7.3–24.7)	0.229
RR8: I have taken drugs and supplies from the hospital to help my patients at my private practice.	64.3	28.6 (11.9–45.3)	0.186	68.3	36.6 (21.9–51.3)	0.029	66.7	33.4 (22.3–44.5)	0.008
RR9: I sometimes have to leave some hours early from work to do my business.	64.3	28.6 (11.9–45.3)	0.186	53.7	7.4 (0.6–15.4)	0.755	58	16.0 (7.3–24.7)	0.229
**RR10: I am sometimes one hour late to work.**	71.4	42.8 (24.5–61.1)	0.038	58.5	17.0 (5.5–28.5)	0.349	63.6	27.6 (17.1–38.1)	0.03

* 2 × Percentage(Y) – 100 (95% confidence interval is shown in parentheses).

**Less sensitive questions are shown in bold type.

The RRT identified three main coping mechanisms that were regularly used. Giving priority to activities that enabled the earning of per diems was the most commonly expressed sensitive earning behavior; it was practiced by 56% of respondents (*p* = 0.000). The other two significant findings were predatory strategies: they involved pilfering drugs from facilities, practiced by 33% of the respondents (*p* = 0.008), and accepting informal payments and gifts from patients in return for priority treatment, which was also practiced by 33% of respondents (*p* = 0.008). These findings confirm the results from the qualitative data. In addition, approximately 30% (p = 0.016) of the respondents reported making more money from supplementary sources than from their salary; this result suggests that health workers who reported earning the equivalent of up to half of their salaries through additional earning arrangements may have underreported that source of income. This is supported by the disparity between the salary and household expenditure of the health workers. It would appear that this difference in income–expenditure can be accounted for by a higher level of supplementary income than was actually reported by health workers. Tables 9 and 10 provide a synthesis of the estimates on these supplementary income sources based on the various information sources obtained in this study. The tables detail the range of income reported by the health workers from their additional medical and non-medical arrangements as well as selling drugs. A combination of these with their official salaries resulted in their reported real income. The tables also detail the deficit between the reported real income and the expenditure of the health workers; thus, they estimate the level of supplementary income that may not have been reported by the respondents. The spousal support reported by some of the respondents was also taken into consideration to enable great accuracy in these estimates.

**Table 9:** Revenues reported by health workers with additional income arrangements in Nasarawa

**CADRES**	**CHEW**		**CHO**		**N/M**		**DOC**	
Level of reticence*	**R**	**Non-R**	**R**	**Non-R**	**R**	**Non-R**	**R**	**Non-R**
Range of income from additional earning arrangements (NGN)	16,200–116,000 (Av: 61,040)	3,000–230,000 (Av: 61,214)	500–112,500 (Av:46,869)	2,000–180,000 (Av:78,327)	54,000–152,500 (Av:116,072)	1,000–196,000 (Av:71,865)	110,000–203,000 (Av:152,667)	3,000–121,500 (Av: 67,791)
Range of income from selling drugs (NGN)	11,000–20,000 (Av: 15,500)	500–80,000 (Av: 15,500)	8,000–10,000 (Av: 9,000)	2,000–30,000 (Av:10,667)	2,000	100,000	0	0
Range of deficit between real income and expenditure (With spousal support) (NGN)	11,900–46,200 (Av:26,367)	18,000–556,000 (Av:197,550)	45,000	92,000–168,000 (AV: 140,143)	42,000–270,000 (Av:163,729)	25,000–230,000 (Av: 107,224)	No deficit	No deficit
Range of deficit between real income and expenditure (Without spousal support) (NGN)	20,000–41,000 (Av: 30,500)	2,800–117,000 (Av:48,450)	46,000–112,585 (Av: 71,003)	64,000–195,000 (Av:130,068)	29,000–464,900 (Av:216,300)	13,000–105,000 (Av:59,000)	−15,000	No deficit

*Reticence is determined by the modified criteria.

NB: Where there is a single figure instead of a range, it indicates that the situation applies to only one health worker in that group.

Av: Average.

NGN: Nigerian Naira.

R: Reticent.

Non-R: Non-reticent.

**Table 10:** Revenues reported by health workers with additional income arrangements in Ondo

**CADRES**	**CHEW**		**CHO**		**N/M**		**DOC**	
Level of reticence*	**R**	**Non-R**	**R**	**Non-R**	**R**	**Non-R**	**R**	**Non-R**
Range of income from additional earning arrangements (NGN)	30,782–65,000 (Av: 53,856)	49,000–98,296 (Av:79,099)	127,500–265,235 (Av:163,213)	10,000–103,070 (Av:81,857)	74,500–200,000 (Av: 104,300)	7,000	15,000–560,000 (Av: 228,667)	500–100,000 (Av:50,250)
Range of income from selling drugs (NGN)	0	0	0	0	0	0	50,000–56,000 (Av: 53,000)	0
Range of deficit between real income and expenditure (With spousal support) (NGN)	53,000–120,000 (73,240)	12,000	206,765– 585,000 (Av: 399,588)	67,860	56,000–286,000 (Av:152,667)	No deficit	3,685,000	No deficit
Range of deficit between real income and expenditure (Without spousal support) (NGN)	40,500–386,433 (Av: 188,383)	431,408	No deficit	55,000	110,000–170,000 (Av:140,500)	No deficit	No deficit	No deficit

*Reticence is determined by the modified criteria.

NB: Where there is a single figure instead of a range, it indicates that the situation applies to only one health worker in that group.

Av: Average.

NGN: Nigerian Naira.

R: Reticent.

Non-R: Non-reticent.

An initial analysis of the RRT data used less restrictive criteria for reticence, and this allowed the behavior of the various types of health workers to be examined. After reticent subjects were excluded, the sample consisted of 145 subjects. When analyzed by type, nurses/midwives (25.8%, *p* = 0.03), CHOs (50%, *p* = 0.00), and CHEWs (59%, *p* = 0.00) prioritized activities that allowed the earning of per diems. Doctors appeared to obtain more income from supplementary sources than from their salaries (23.6%, *p* = 0.05). Significantly more CHEWs (7.6%, *p* = 0.05) reported accepting money and gifts from patients in return for priority treatment, whereas taking drugs and supplies from facilities to treat patients in private practice was a significant result only for CHOs (16.6%, *p* = 0.05).

Nurses/midwives showed the greatest reticence (34%, n = 32), whereas CHOs showed the least (20%, n = 19). These differences among health worker type were statistically significant (*p* < 0.05). This difference in the distribution of reticence could be a possible reason for the differences observed in additional earning arrangements among states and health worker types. The sensitive coping strategies of the various types of health workers as assessed using the RRT are shown in Additional file 1: Table S3.

## Discussion

The study explored the official remunerations of health workers in Nigeria and the opinions of health workers and policy makers about its adequacy to maintain a reasonable standard of living. A disparity in views was found between the two groups. Health workers’ household expenditure patterns were also examined along with their coping strategies; the results offer valuable insight into the dynamics of such workers’ responses to income-related issues in the two Nigerian states where the investigation took place. Revenues from the health workers’ coping mechanisms were estimated, and in combination with expenditure patterns, these provided some insight into the real income of health workers.

Within the context of the Nigerian public service, the official remuneration of health workers is regarded as favorable, as they are considered the best-paid civil servants. For example, doctors earn more than three times the salaries of their counterparts in the public service. In their study on the adequacy of health workers’ salaries in Ghana, Witter et al. [21] showed that health workers’ salaries were better than those of general civil service employees. This is in keeping with the findings of Bowie et al. [22] in their study in Malawi, where they determined that the salaries of health workers were 52% higher than their counterparts in the public service.

The health workers in the study population held differing opinions about the adequacy of their wages. Most workers considered their salaries insufficient, and the level of dissatisfaction with their salaries and benefits was notably at variance with the views of key informants; this disparity may be the result of societal perceptions of the health workers’ privileged position. It also became clear that irrespective of whether CONMESS and CONHESS were implemented at 100% or 80% of the federal scale, the level of dissatisfaction among the health workers in the study populations was similar. The prime issue with regard to dissatisfaction thus may not be related to salary level. The expenditure–income asymmetry observed in this study demands closer scrutiny—especially with regard to the proportion of the health workers’ income reportedly spent on household feeding, children’s school fees, and on other dependents. Spousal support, which was reported by a number of respondents, cannot be discounted: 44% (n = 73) of the health workers had spouses in full-time employment. However, the expenditure–income disparity may explain the general level of dissatisfaction that the health workers felt with regard to their general living conditions.

A key role of personal income is to cater for the needs of one’s family—whether nuclear or extended. For the majority of health workers in this study, household feeding accounted for a considerable portion of their salaries, with almost 20% of them spending more than half of their salaries in that area. This finding may be explained by the relatively large sizes of some of the households surveyed and, of course, the cost of living in Nigeria. The value the respondents placed on their extended families was evident in the proportion of the health workers’ salaries assigned to this area. Over 20% of respondents apportioned between 21% and 50% of their salaries to their dependents. The perceived pressure on the part of the health workers became clear in the interviews. The extended family culture is deeply embedded in African society, and the attendant obligations are widely embraced and rarely questioned. However, in addition to the extended family culture, the high social expectations of health workers—as a result of the perceived prestigious nature of their jobs—may further complicate this spending pattern. There is a lack of research in this area, and so it would be interesting to compare the expenditure pattern of health workers with that of public servants in other sectors.

Examining the expenditure patterns of health workers in this study provided some insight into what drives them to supplement their income despite receiving salary increases. Given health workers’ perceived needs, it is clear that the salary and allowances earned in the public health sector are insufficient. This may remain the case irrespective of increases in salary and allowances. It is therefore difficult—if not impossible—to prevent these workers from making additional earning arrangements. Consequently, it is necessary to regulate those activities. The main option could be properly designed accountability mechanisms to regulate the amount of time spent on non-predatory activities, and thus ensure that they do not conflict with official work. This would be in line with the findings of Bowie et al. [22], who determined that health workers’ basic salaries in Malawi were insufficient to reduce the incentive to make additional earning arrangements. Similarly, in their Tanzanian study, Stringhini et al. [23] found that despite a salary increase of 70% for doctors and 30% for nurses, health workers still considered their salaries inadequate in terms of their needs and workload.

The value of triangulating the answers of respondents by means of mixed methods became evident in this study. The RRT was used to identify and remove the reticent group in the study population, and it enabled an analysis of participants who were more candid about their coping mechanisms to supplement their incomes. By means of the RRT, both non-predatory and predatory activities were clearly identified as reported by the non-reticent respondents. In both states, giving priority to activities that enabled the earning of per diems emerged as the most prevalent coping mechanism; however, the information from the survey and interviews indicated that there may not be many opportunities for this to take place. Predatory mechanisms, such as accepting money or gifts from patients in return for priority treatment and stealing drugs and supplies from facilities to treat private patients, were also significant findings in this study. These findings are in line with those of Macpake et al. [11] in their study of coping strategies among health workers in Uganda, where drug leakage was a major source of revenue for health workers and their managers. Similarly, Stringhini et al. [23] found informal payments collected from patients to be an important source of income for health workers. In the present study, the ability to make more than one’s official salary from supplementary sources was also a significant finding among non-reticent health workers. This finding is in line with that in a study cited by Van Leberghe et al. [7], in which additional earning arrangements more than doubled the median income of health managers. This result further stresses the value of extra earning arrangements in allowing health workers to live close to their desired level of comfort. The implication of additional income earning activities in PBF proves the necessity of ensuring that vigilant monitoring activities are in place to discourage illegal practices.

The dynamics in the two states varied to some extent: the two population groups were perceptibly different in terms of their openness in providing answers, both in the survey and in the interviews. The general impression of acceptability of some of these sensitive practices in Nasarawa may partly explain this diversity. However, enabling environments also appear to have contributed to the differences. For example, in Nasarawa, there was a lack of functioning DRFs in many facilities, as reported by the health workers and key informants: this creates opportunities for illegal practices. Ondo’s free health-care may have had the opposite effect of reducing the opportunities for such practices—at least for the population groups that had access to it. The same cannot be said for the rest of the population.

### Study limitations

It is highly likely that the underreporting of medical-related earning arrangements occurred in this study, especially with regard to predatory activities that occurred during work hours. It is not clear how on-call services impacted the normal work of health workers in this study; such services can operate over periods of up to 24 hours a day. If an emergency occurred when health workers were on call while engaged in private practice, this could mean that they could not attend their regular workplaces. It is possible that the non-medical business practiced by 42.4% of the respondents was also underreported. The issue of private practice was quite a sensitive one for many respondents, especially in the interview setting. One reason for this could be that only doctors were identified as having official permission to undertake private practice outside of regular work hours. However, to a large extent, this did not appear to encourage the doctors to report such activities. Many professed to be too busy with their official work for private practice, but they were willing to talk about their colleagues who engaged in those practices.

A combination of methods was used to tackle the sensitive subject of health workers’ coping strategies. The reticence evident among health workers and key informants in describing these activities despite the variety of investigative tools employed indicates the challenges in examining such sensitive issues. The health workers were willing to participate in this study, but their apparent distrust in the system resulted in them not being forthcoming about their coping strategies even when they had been assured of confidentiality. Some health workers were interviewed outside their facilities and some were interviewed within, but that did not seem to make a difference. A further limitation of this study is the fact that it was the health workers’ managers that put their names forward for this survey, and encouraged them to participate. Convenience sampling was used for CHOs in Ondo and doctors in Nasarawa, which means that some of those not recruited into the study may have been absent because of additional earning arrangements. There was no discernible difference in the analysis between those health workers who were randomly sampled and those recruited by convenience sampling.

## Conclusions

This study shows that health workers’ salaries are perceived as being inadequate to meet their needs because of considerable expenditure–income asymmetry. The income accrued through coping strategies to supplement workers’ salaries was both substantial and necessary in view of the high disparity. However, it may be difficult to fully meet the health workers’ expenses through their salaries and financial incentives, which emphasizes the need for the regulation of additional earnings and targeted accountability mechanisms.

The additional earning arrangements of health workers comprised both predatory and non-predatory activities. The difficulty in eliciting information both in the survey and interviews because of reticence by the study population shows that the predatory behaviors identified in this study were only indicative. Such non-predatory strategies as farming and trading can be regulated to ensure that they do not conflict with the official work hours of health workers; however, predatory mechanisms impede access to and the quality of health-care, and they need to be dealt with more rigorously. In general, addressing these strategies involves putting in place properly designed accountability mechanisms, which in turn requires building capacity in human resource management. In essence, performance management structures have to be implemented to ensure efficiency.

This study displays the value of using mixed methods in investigating sensitive issues. More studies of this type need to be carried out using mixed methods for triangulation purposes to provide better insight into problems in the health sector.

## Endnotes

^a^All of the tertiary health facilities in both states offered specialized care but none was a teaching hospital.

^b^It is usually used in surveys dealing with sensitive issues, including drug use and cheating [1]. Many potential respondents in research investigating sensitive issues refuse to answer or provide false answers. With the RRT, because their answers are guaranteed to be private, respondents are supposed to be more encouraged to answer truthfully, but the RRT has had limited success in achieving this. Its main value, however, is in identifying those who give improbable answers; that is, the reticent respondents [2]. The procedure:

Step 1: The respondent was given a coin as the chosen randomization device.

Step 2: The respondent was faced with a sensitive question.

Step 3: The respondent flipped the coin without showing it to the interviewer; if the coin landed on heads then the respondent was supposed to answer “yes” to the question. If the coin landed on tails, then the respondent was to answer truthfully to the question.

^1^Londino G, Waung C. How to ask sensitive questions using statistics: a case study of academic dishonesty. B.S Undergraduate Mathematics Exchange. 2004;2:1.

^2^Omar A, Murrell P. Identifying Reticent Respondents: Assessing the Quality of Survey Data on Corruption and Values. Economic Development and Cultural Change, University of Chicago Press. 2009;57:2.

^c^At the time of the study, the official exchange rate for 1 dollar was 154 Naira.

## Abbreviations

CHEW: Community health extension worker; CHO: Community health officer; CONHESS: Consolidated health salary structure; CONMESS: Consolidated medical salary structure; HRH: Human resources for health; IMR: Infant mortality rate; LGA: Local government area; LMICs: Low and middle-income countries; NPHCDA: National primary health care development agency; PBF: Performance-based financing; RBF: Results-based financing; RRT: Randomized response technique; SPSS: Statistical package for the social sciences.

## Competing interests

The authors declare that they have no competing interests.

## Authors’ contributions

NA, DS, CL, and MD all made substantial contributions to the study design, interpretation of findings and critical revision of drafts. NA carried out the fieldwork. DS conducted the statistical analysis. NA, DS, and MD contributed to the analysis of the findings and the drafting of the manuscript. All authors read and approved the final manuscript.

## Authors’ information

Ngozi Akwataghibe MBBS, MPH is a public health expert and an experienced project manager with a focus on human resources for health management research at ENAULD Health Research, The Netherlands. At the time of this study, she worked as a consultant for the Royal Tropical Institute, The Netherlands.

Dulani Samaranayake MBBS, MSc, MD is a specialist in community medicine with a special interest in human resources for health and occupational health. She is currently attached to the Faculty of Medicine, University of Colombo as a lecturer.

Christophe Lemiere, MPH, MBA is a senior health specialist at the World Bank. Within this institution, he leads the World Bank African program on Human Resources for Health.

Marjolein Dieleman MA, MPH, PhD is a specialist in human resources for health, a senior public health expert, and coordinator of the WHO Collaborating Center for Research, Training and Development of Human Resources for Health at the Royal Tropical Institute, Amsterdam.

## Pre-publication history

The pre-publication history for this paper can be accessed here:

http://www.biomedcentral.com/1472-6963/13/387/prepub

## Supplementary Material

Additional file 1: Table S1 Total household expenditure as a percentage of the salary of the health workers. **Table S2.** Selling drugs as an additional earning arrangement. **Table S3.** Prevalence of sensitive coping strategies as assessed by the RRT according to health worker type. Click here for file
