# Supplementary material for: Assessing health workers’ revenues and coping strategies in Nigeria — a mixed-methods study
Source: BMC Health Serv Res. 2013 Oct 4;13:387. doi: 10.1186/1472-6963-13-387 (PMC3853328; doi:10.1186/1472-6963-13-387)
Supplement: Additional file 1: Table S1 — Total household expenditure as a percentage of the salary of the health workers. Table S2. Selling drugs as an additional earning arrangement. Table S3. Prevalence of sensitive coping strategies as assessed by the RRT according to health worker type. [file 1472-6963-13-387-S1.docx]

# Additioanl file 1

**Table S1: Total household expenditure as a percentage of the salary of the health workers**

| Percentage of salary | No of health workers | Percentage |
| --- | --- | --- |
| 50 or less | 18 | 10.9 |
| 51 - 100 | 39 | 23.6 |
| 101 - 200 | 62 | 37.6 |
| 201 - 300 | 21 | 12.7 |
| 301 - 500 | 12 | 7.3 |
| >500 | 8 | 4.8 |
| Total | 160 | 97.0 |
| Missing system | 5 | 3.0 |
| Total | 165 | 100 |

**Table S2: Selling drugs as an additional earning arrangement**

| **Practices and earnings** | **By self** | | **By others** | |
| --- | --- | --- | --- | --- |
|  | Frequency | Percentage | Frequency | Percentage |
| Selling drugs |  |  |  |  |
| Yes | 25 | 15.2 | 50 | 30.3 |
| No | 135 | 81.8 | 112 | 67.9 |
| Not stated | 05 | 3.0 | 03 | 1.8 |
|  |  |  |  |  |
| Amount earned from selling drugs* |  |  |  |  |
| <10000 | 11 | 44.0 | 0 | 0.0 |
| 10000 to 50000 | 08 | 32.0 | 13 | 26.0 |
| 50000 to 100000 | 03 | 12.0 | 09 | 18.0 |
| >100000 | 0 | 0.0 | 06 | 12.0 |
| Not specified | 03 | 12.0 | 22 | 44.0 |

*Out of the 25 who claimed to sell drugs and out of the 50 who claimed that others sell drugs

**Table S3: Prevalence of sensitive coping strategies as assessed by the RRT according to health worker type**

NB- Reticence is determined by the standard criteria

*Binomial Test

**Less sensitive questions are shown in bold type.
